# Knowledge About the Herpes Zoster (HZ) Vaccine and Its Acceptance Among the Population in Al-Ahsa City in the Kingdom of Saudi Arabia

**DOI:** 10.7759/cureus.50329

**Published:** 2023-12-11

**Authors:** Abdullah H Bohamad, Heba Y Alojail, Lujain A Alabdulmohsin, Meshari A Alhawl, Mishal B Aldossary, Fatimah M Altoraiki, Abdullatif Y Almulhim, Abdullah Almulhim, Shahd Alabdulathim, Fatimah Almarri

**Affiliations:** 1 Dermatology, King Faisal University, Al-Ahsa, SAU

**Keywords:** acceptance vzv, acceptance, vaccination, vzv, vaccine, vzv vaccine

## Abstract

Introduction

Herpes zoster (HZ), caused by the varicella-zoster virus (VZV), poses a public health concern in Saudi Arabia, with an increasing number of cases reported in recent years. This study aimed to assess the knowledge and acceptance of the herpes zoster vaccine among Saudi Arabian citizens in Al-Ahsa.

Methodology

A descriptive cross-sectional study was conducted using online surveys administered via social media platforms. The study was conducted in Al-Ahsa, a city located in the Eastern Province of Saudi Arabia. Al-Ahsa serves as a representative urban area within Saudi Arabia.

Result

The study found a high level of awareness and knowledge about the vaccine, with 78.2% of the participants having heard of herpes zoster. The majority considered the vaccine effective (89%) and believed it was available in Saudi Arabia (87%). However, the vaccination rate was only 8%. The study revealed varied opinions on the target population for vaccination, with 39.7% favoring immunocompromised patients and 38.1% choosing individuals above 50 years. The reasons for not being vaccinated included a lack of information (38.3%) and the belief of being in good health (37.3%).

Conclusion

The findings suggest a need for targeted educational campaigns to address misconceptions and promote vaccination, particularly among healthcare providers and the recommended target populations. Future research should explore barriers to vaccination to inform tailored interventions.

## Introduction

The varicella-zoster virus (VZV), responsible for chicken pox, can later reactivate in life, causing herpes zoster (HZ), commonly known as shingles, affecting individuals of any age. Also, VZV can lead to complications such as pneumonia, encephalitis, and postherpetic neuralgia. Vaccination, particularly the varicella-zoster vaccine, has significantly decreased the incidence of these complications. Herpes zoster is more prevalent in those over 50 and immunocompromised patients [1]. Globally, the incidence of herpes zoster varies across different age groups. Among healthy, young individuals, the annual occurrence ranges from 1.2 to 3.4 cases per 1000 individuals. However, in adults, there is an increased prevalence compared to the younger population. Notably, among those over 50 years, herpes zoster becomes more prevalent, and individuals above 65 years experience a further escalation in incidence, ranging from 3.9 to 11.8 cases per 1000 individuals annually [2]. This age-stratified information underscores the impact of age on the prevalence of herpes zoster, with a notable increase in the risk among individuals over 50 years old and particularly those above 65 years old. One such vaccine is the herpes zoster vaccine, designed to mitigate the risk and severity of herpes zoster and its associated complications [3]. Similar to many other countries, Saudi Arabia has witnessed an escalating incidence of herpes zoster cases, raising public health concerns [4]. Numerous studies underscore the necessity for educational interventions to enhance awareness and knowledge about herpes zoster vaccination [5,6]. Additionally, prior research has underscored the influence of demographic factors, including age, gender, and education, on vaccine acceptance [7,8]. Hence, it is imperative to investigate the awareness, knowledge, and acceptance of the herpes zoster vaccine among Saudi Arabian citizens residing in Al-Ahsa. The study aimed to assess the knowledge regarding the HZ vaccine and its acceptance among the people in Al-Ahsa, Saudi Arabia.

## Materials and methods

Study design

The study employed a descriptive cross-sectional design and employed a population-based survey administered through online questionnaires. These questionnaires were distributed using social media platforms to investigate the awareness, knowledge, and acceptance of the herpes zoster vaccine among Saudi Arabian citizens residing in Al-Ahsa.

Study area and settings

The study was conducted in Al-Ahsa, situated in the Eastern Province of Saudi Arabia, serving as a representative urban area within the country. The research targeted the general population of Al-Ahsa, employing an online survey platform and social media channels for participant recruitment. This method capitalized on the extensive use of social media platforms such as WhatsApp, Instagram, Snapchat, and Twitter among Saudi Arabian citizens, ensuring a convenient and efficient data collection process. All surveys and questionnaires were administered online, providing participants with the flexibility to complete them at their convenience and from the comfort of their homes. The online modality enhanced accessibility and outreach, thereby increasing the likelihood of obtaining a representative sample from the intended population.

Study population

The population of interest encompassed individuals aged 18 years and older. The inclusion criteria comprised Saudi Arabian citizenship, age of 18 years and above, and residency in Al-Ahsa. The exclusion criteria encompassed non-Saudi citizens and individuals under the age of 18 years.

The data collection and analysis

The data collection instrument comprised a structured questionnaire tailored for this study. We conducted a comprehensive literature review on the questionnaire, crafted our survey, and sought face validity from two experts. Following the validation, we carried out a pilot study involving 25 participants. The questionnaire was systematically divided into sections to evaluate awareness, knowledge, and acceptance of the herpes zoster vaccine. It incorporated close-ended questions, encompassing multiple-choice and yes/no formats, designed to elicit quantitative data. The data derived from the online questionnaires underwent analysis utilizing Statistical Package for Social Sciences (SPSS) (IBM SPSS Statistics, Armonk, NY), a robust statistical software platform, to achieve the objectives of the study.

Research ethics approval

The research proposal was submitted to the Research Ethics Committee at King Faisal University for comprehensive ethical review and approval prior to the initiation of the study, with approval number KFU-REC-2023-NOV-ETHICS1362.

## Results

In Table 1, the comprehensive demographic overview reveals that 487 participants were surveyed. The gender distribution indicates that 288 (59.1%) were females and 199 (40.9%) were males. Agewise, 54% were between 20 and 35 years, 19.5% 36-50 years, 15.6% over 50 years, and 10.9% under 21 years. Regarding marital status, 54.2% were married, 43.1% single, 1.4% divorced, and 1.2% widowed. Education levels varied, with 58.7% holding a bachelor's degree, 17.7% completing high school, and smaller percentages having other educational backgrounds. As for employment status, 39.7% were employed, 33.7% students, 12.7% retired, 6.8% housewives, and 7.4% unemployed. Monthly income distribution ranged from less than 300 Saudi riyal (SR) to more than 20000 SR. This detailed demographic breakdown provides a comprehensive understanding of the surveyed population.

**Table 1:** Demographic characteristics SR: Saudi riyal

Variables	Categories	N	%
Gender	Male	199	40.9
	Female	288	59.1
Age	Less than 21 years	53	10.9
	From 21 to 35 years	263	54
	From 36 to 50 years	95	19.5
	More than 50 years	76	15.6
Marital status	Single	210	43.1
	Married	264	54.2
	Divorced	7	1.4
	Widowed	6	1.2
Education	Primary	4	0.8
	Intermediate	9	1.8
	High school	86	17.7
	Diploma	80	16.4
	Bachelor	286	58.7
	Postgraduate	22	4.5
Occupation status	Student	164	33.7
	Employed	192	39.4
	Housewife	33	6.8
	Unemployed	36	7.4
	Retired	62	12.7
Income	Less than 3000 SR	183	37.6
	From 3000 to 10000 SR	150	30.8
	From 10000 to 20000 SR	111	22.8
	More than 20000 SR	43	8.8

In Table 2, the data highlights that a substantial 78.2% of the participants were aware of herpes zoster. Additionally, 4.5% reported personal experiences with the disease, while 43.9% knew someone who had encountered it. Notably, 38% of the surveyed individuals had received information about the herpes zoster vaccine. These percentages provide insights into the varying levels of awareness, personal experiences, and connections to herpes zoster within the studied population.

**Table 2:** Knowledge about the herpes zoster vaccine

Number	Question	Yes	No
1	Have you heard of the disease?	381	106
		78.2%	21.8%
2	Have you ever had the disease?	22	465
		4.5%	95.5%
3	Do you know someone who has had herpes zoster (HZ)?	214	273
		43.9%	56.1%
4	Have you ever received any information related to the herpes zoster vaccine?	185	302
		38%	62%

In Table 3, the data reveals that the Internet served as the primary source of information about the VZV vaccine for 30.5% of the respondents, followed closely by books at 27.8%. Health practitioners were a significant source at 24.5%, while TV, university studies, general health promotion, and information from friends/relatives contributed to varying degrees. Infected cases constituted the least common source at 1.7%. These percentages provide insights into the diverse channels through which individuals gathered information about the VZV vaccine.

**Table 3:** Sources of information about the VZV vaccine VZV: varicella-zoster virus

Number		N	%
1	Internet	92	30.5
2	Health practitioners	74	24.5
3	Books	84	27.8
4	TV	19	6.3
5	General health promotion	9	3
6	Friends, relatives, and family	9	3
7	University studies	10	3.3
8	Infected cases	5	1.7

In Table 4, the findings indicate strong beliefs in the effectiveness of the VZV vaccine, with 89% of the respondents expressing confidence. Additionally, 87% believed that the vaccine was available in the Kingdom of Saudi Arabia (KSA). Only 8% of the participants reported being vaccinated with the VZV vaccine. A considerable 75% expressed a willingness to recommend the vaccine to friends or relatives. Interestingly, nearly half, specifically 48%, favored vaccination even if payment was required. These percentages offer insights into the perceived effectiveness, accessibility, and acceptance of the VZV vaccine within the surveyed population.

**Table 4:** Acceptance of the herpes zoster (HZ) vaccine VZV, varicella-zoster virus; KSA, Kingdom of Saudi Arabia

Number	Question	Yes	No
1	Is the VZV vaccine effective?	432	55
		89%	11%
2	Is the VZV vaccine available in the KSA?	423	64
		87%	13%
3	Have you been vaccinated with the HZ vaccine?	37	450
		8%	92%
4	Would you recommend taking the HZ vaccine for a friend or relative?	363	124
		75%	25%
5	Are you in favor of vaccination against HZ even if you must pay?	232	255
		48%	52%

In Table 5, the data reveals that 39.7% of the respondents believed that immunocompromised patients should be vaccinated with the VZV vaccine. Additionally, 38.1% expressed the opinion that individuals above 50 years should receive the vaccine. A smaller percentage, 2.6%, believed in neither category, while 19.6% admitted to not knowing. These figures provide insights into the varying perspectives on target demographics for the VZV vaccine among the surveyed population.

**Table 5:** Who should be vaccinated with the VZV vaccine VZV: varicella-zoster virus

Number	Items	N	%
1	People above 50	250	38.1
2	Immunocompromised patient	261	39.7
3	None of the above	17	2.6
7	I do not know	129	19.6

In Table 6, the data highlights that the primary reason for the unwillingness to be vaccinated was a lack of information, with 38.3% expressing this concern. Subsequently, 37.3% attributed their reluctance to a belief of being well. Other reasons included concerns about vaccination risks (11.9%), perceiving it as not beneficial (5.4%), financial constraints (3.8%), the belief that it is for elderly people (2.4%), and a previous infection (0.9%). These percentages shed light on the diverse factors influencing individuals' decisions against vaccination.

**Table 6:** Reasons for not willing to be vaccinated

Number	Items	N	%
1	Belief that we are well	206	37.3
2	Lack of information	212	38.3
3	Consider vaccination too risky	66	11.9
4	Inability to afford	21	3.8
5	Thinking it is not beneficial	30	5.4
6	A previous infection	5	0.9
7	Belief that it is for elderly people	13	2.4

In Table 7, the results indicate a noteworthy association between gender and knowing someone with HZ, with females at 69.6% and males at 30.4% (χ² = 17.379; P < 0.001). Similarly, a significant association is observed concerning receiving information about the HZ vaccine, where females (66.5%) exceeded males (33.5%) (χ² = 6.667; P = 0.010). However, no significant associations were found regarding hearing about HZ and having HZ. These statistical findings underscore gender-related disparities in awareness and information uptake regarding herpes zoster within the surveyed population.

**Table 7:** The association between knowledge items and gender *Significant at 0.05

Yes	Male	Female	Chi-square	P-value
Have you heard of the disease?	150	231	1.613	0.204
	39.4%	60.6%		
Have you ever had the disease?	9	13	0	0.966
	40.9%	59.1%		
Do you know someone who has had HZ?	65	149	17.379	<0.001*
	30.4%	69.6%		
Have you ever received any information related to the HZ vaccine?	62	123	6.667	0.010*
	33.5%	66.5%		

In Table 8, the results reveal a significant association between the age and awareness of HZ (χ² = 48.354; P < 0.001). Notably, the age group of 21-35 years constitutes the largest percentage, with 52.8% having heard of HZ (χ² = 16.799; P < 0.001). Furthermore, the age group of more than 45 years represents the largest percentage, with 40% (nine individuals) who knew someone with HZ (χ² = 44.1; P < 0.001). However, there is no significant association observed among those who have ever received information related to the VZV vaccine. These findings highlight age-related patterns in herpes zoster awareness within the surveyed population.

**Table 8:** The association between knowledge items and age *Significant at 0.05 HZ: herpes zoster

Yes	Less than 20 years	From 21 to 35 years	From 36 to 50 years	More than 50 years	Chi-square	P-value
Have you heard of the disease?	25	201	82	73	48.354	<0.001*
	6.6%	52.8%	21.5%	19.2%		
Have you ever had the disease?	0	6	7	9	16.799	<0.001*
	0%	27.3%	31.8%	40%		
Do you know someone who has had HZ?	13	92	62	47	44.1	<0.001*
	6.10%	43%	29%	22%		
Have you ever received any information related to the HZ vaccine?	16	98	34	37	5.313	0.150
	8.6%	53%	18.4%	20%		

In Table 9, the results demonstrate a significant association between marital status and the awareness of HZ (χ² = 23.681; P < 0.001), with married participants constituting the largest percentage at 60%. Similarly, there is a significant association between marital status and knowing someone with HZ (χ² = 23.626; P < 0.001), where married participants represent the largest percentage at 77% (17 individuals). However, no significant associations are found with having HZ or receiving any information related to the VZV vaccine. These findings underscore the impact of marital status on awareness and personal connections to herpes zoster within the surveyed population.

**Table 9:** The association between knowledge items and marital status *Significant at 0.05 HZ: herpes zoster

Yes	Single	Married	Divorced	Widowed	Chi-square	P-value
Have you heard of the disease?	144	228	4	5	23.681	<0.001*
	38%	60%	1%	1%		
Have you ever had the disease?	5	17	0	0	5.098	0.165
	23%	77%	0%	0%		
Do you know someone who has had HZ?	67	140	5	2	23.626	<0.001*
	31%	65%	2%	1%		
Have you ever received any information related to the HZ vaccine?	79	100	2	4	2.372	0.499
	43%	54%	1%	2%		

In Table 10, the results highlight a significant association between education and having HZ (χ² = 18.112; P = 0.003), with the largest percentage (64%) observed among those with a bachelor's degree. Similarly, a significant association is found between education and receiving information related to the HZ vaccine (χ² = 11.43; P = 0.043), where the largest percentage (63%) is among those with a bachelor's degree. However, no significant associations are identified with hearing about HZ or having HZ. These findings suggest an educational influence on the prevalence of herpes zoster and the acquisition of information about the associated vaccine within the surveyed population.

**Table 10:** The association between knowledge items and education *Significant at 0.05 VZV, varicella-zoster virus; HZ, herpes zoster; KSA, Kingdom of Saudi Arabia

Yes	Male	Female	Chi-square	P-value
Is the VZV vaccine effective?	175	257	0.197	0.657
	41%	59%		
Is the VZV vaccine available in the KSA?	168	255	1.750	0.186
	40%	60%		
Have you been vaccinated with the HZ vaccine?	23	14	7.518	0.006*
	62%	38%		
Would you recommend taking the HZ vaccine for a friend or relative?	151	212	0.319	0.572
	42%	58%		
Are you in favor of vaccination against HZ even if you must pay?	90	142	0.785	0.376
	39%	61%		

In Table 11, the results reveal a significant association between gender and the VZV vaccine (χ² = 7.518; P = 0.006), indicating that 62% of males and 38% of females have been vaccinated. However, there is no significant association found regarding opinions on the effectiveness of the VZV vaccine, its availability in the KSA, willingness to recommend the VZV vaccine, or support for vaccination against VZV even with the associated costs. These findings highlight gender-related differences specifically concerning herpes zoster vaccination within the surveyed population.

**Table 11:** The association between acceptance items and gender *Significant at 0.05 VZV, varicella-zoster virus; HZ, herpes zoster; KSA, Kingdom of Saudi Arabia

Yes	Male	Female	Chi-square	P-value
Is the VZV vaccine effective?	175	257	0.197	0.657
	41%	59%		
Is the VZV vaccine available in the KSA?	168	255	1.750	0.186
	40%	60%		
Have you been vaccinated with the HZ vaccine?	23	14	7.518	0.006*
	62%	38%		
Would you recommend taking the HZ vaccine for a friend or relative?	151	212	0.319	0.572
	42%	58%		
Are you in favor of vaccination against HZ even if you must pay?	90	142	0.785	0.376
	39%	61%		

In Table 12, the results highlight a significant association between age and the perception of the effectiveness of the VZV vaccine (χ² = 9.0454; P = 0.0029), with the age group of 21-35 years representing the largest percentage at 56% (130 individuals). Furthermore, there is a significant association between age and recommending the VZV vaccine for a friend or relative (χ² = 11.491; P = 0.0009), where the age group of 21-35 years accounts for the largest percentage at 58%. However, no significant associations are found with individuals who have been vaccinated with the VZV vaccine, those who believe that the VZV vaccine is available in the KSA, and those in favor of the VZV vaccine even if they must pay. These findings emphasize age-related variations in perceptions and recommendations concerning VZV vaccine within the surveyed population.

**Table 12:** The association between acceptance items and age *Significant at 0.05 VZV, varicella-zoster virus; HZ, herpes zoster; KSA, Kingdom of Saudi Arabia

Yes	Less than 20 years	From 21 to 35 years	From 36 to 50 years	More than 50 years	Chi-square	P-value
Is the VZV vaccine effective?	47	243	79	63	9.0454	0.029*
	11%	56%	18%	15%		
Is the VZV vaccine available in the KSA?	42	230	87	64	5.094	0.165
	10%	54%	21%	15%		
Have you been vaccinated with the HZ vaccine?	4	24	5	4	2.202	0.532
	11%	65%	14%	11%		
Would you recommend taking the HZ vaccine for a friend or relative?	38	209	70	46	11.491	0.009*
	10%	58%	19%	13%		
Are you in favor of vaccination against HZ even if you must pay?	25	130	46	31	1.795	0.616
	11%	56%	20%	13%		

In Table 13, the results reveal a significant association between marital status and the perception of the effectiveness of the VZV vaccine (χ² = 14.093; P = 0.0003), with the largest percentage (56%) among married participants. However, no significant associations are found with individuals who have been vaccinated with the VZV vaccine, those who would recommend the VZV vaccine for a friend or relative, those with the belief in the availability of the VZV vaccine in the KSA, and those in favor of the VZV vaccine even if they must pay. These findings suggest that marital status may influence specific perceptions related to the effectiveness of the VZV vaccine within the surveyed population.

**Table 13:** The association between acceptance items and marital status *Significant at 0.05 VZV, varicella-zoster virus; HZ, herpes zoster; KSA, Kingdom of Saudi Arabia

Yes	Single	Married	Divorced	Widowed	Chi-square	P-value
Is the VZV vaccine effective?	195	228	6	3	14.093	0.003*
	45%	53%	1%	1%		
Is the VZV vaccine available in the KSA?	182	230	6	5	2.102	0.992
	43%	54%	1%	1%		
Have you been vaccinated with the HZ vaccine?	21	16	0	0	3.684	0.298
	57%	43%	0%	0%		
Would you recommend taking the HZ vaccine for a friend or relative?	166	189	5	3	5.398	0.145
	46%	52%	1%	1%		
Are you in favor of vaccination against HZ even if you must pay?	103	123	5	1	4.179	0.243
	44%	53%	2%	0%		

In Table 14, the results indicate that there is no significant association between education and all acceptance items. This suggests that, within the surveyed population, education does not appear to play a significant role in influencing acceptance regarding the items under consideration.

**Table 14:** The association between acceptance items and education VZV, varicella-zoster virus; HZ, herpes zoster; KSA, Kingdom of Saudi Arabia

Yes	Primary	Intermediate	High school	Diploma	Bachelor	Postgraduate	Chi-square	P-value
Is the VZV vaccine effective?	3	8	76	69	256	20	1.533	0.909
	1%	2%	18%	16%	59%	5%		
Is the VZV vaccine available in the KSA?	3	7	74	69	252	18	2.102	0.835
	1%	2%	17%	16%	60%	4%		
Have you been vaccinated with the HZ vaccine?	0	1	4	1	27	4	11.036	0.051
	0%	3%	11%	3%	73%	11%		
Would you recommend taking the HZ vaccine for a friend or relative?	3	6	62	54	221	17	3.867	0.569
	1%	2%	17%	15%	61%	5%		
Are you in favor of vaccination against HZ even if you must pay?	1	5	38	36	138	14	3.983	0.552
	0%	2%	16%	16%	59%	6%		

## Discussion

The findings of this study illuminate the awareness, knowledge, and acceptance of herpes zoster (HZ) vaccination among Saudi Arabian citizens in Al-Ahsa, providing valuable insights for public health interventions. Additionally, the study aimed to compare the level of acceptance and awareness of HZ vaccination with existing literature.

More than three-quarters of participants were aware of herpes zoster, while only a small percentage had experienced the disease. The study observed a high acceptance rate, with participants believing that the target population for the varicella-zoster virus (VZV) vaccine should include individuals above 50 years, followed by immunocompromised individuals. The most common reasons for the unwillingness to be vaccinated were a lack of information and the belief of being well.

The results indicated that a majority of the participants had heard of herpes zoster, albeit less than reported in a previous study conducted in Saudi Arabia [9]. In terms of the acceptance of the herpes zoster vaccine, a high percentage believed the VZV vaccine to be effective (89%) and available in Saudi Arabia (87%). However, the vaccination rate (8%) was lower than expected. This aligns with a small percentage of individuals who had already been vaccinated, consistent with a previous study conducted in Saudi Arabia [9]. This similarity may be attributed to factors such as accessibility or vaccine availability in both study populations.

Regarding the target population for the VZV vaccine, a significant proportion believed that immunocompromised patients (39.7%) and individuals above 50 years of age (38.1%) should be vaccinated, aligning with previous research conducted in Italy [10]. However, a small percentage believed that none of these groups should receive the vaccine. The lack of unanimous agreement on the target population indicates the need for further education and awareness campaigns to provide evidence-based recommendations. The primary reasons for reluctance to be vaccinated were predominantly attributed to a lack of information (38.3%) and a belief of being well (37.3%). These findings are consistent with previous studies conducted in other countries [11].

Limitations and recommendations

The study included a relatively small sample size, which may limit the generalizability of the findings to the entire Saudi Arabian population. A larger sample size would have provided more robust and representative results. The participants were recruited from a specific region in Saudi Arabia (Al-Ahsa), which may introduce selection bias. The findings of this study may not be applicable to other areas or populations within Saudi Arabia. The data collected in this study was self-reported by the participants, which may be subject to recall bias or social desirability bias. The participants may have provided responses that they perceived as more socially acceptable or may have had difficulty accurately recalling certain information. The use of social media platforms, including Snapchat, Twitter, and WhatsApp, for data collection poses challenges regarding data accuracy and reliability. Researchers should be mindful of potential biases associated with these platforms. Ensuring data precision is essential for maintaining research integrity. Strategies should be employed to address these challenges and enhance the overall quality of study outcomes.

The study utilized a cross-sectional design, which only captures a snapshot of the participants' knowledge, awareness, and acceptance of herpes zoster vaccination at a single point in time. This design does not allow for the evaluation of temporal relationships or changes in attitudes and behaviors over time. The study focused on a specific region in Saudi Arabia (Al-Ahsa). The cultural, social, and economic factors within this region may differ from other regions in the country, limiting the generalizability of the findings beyond this specific context. As this study was conducted in Saudi Arabia, the findings may not be applicable to other countries or populations with different healthcare systems, cultural beliefs, and healthcare utilization patterns. To enhance the reliability and generalizability of future studies, researchers may consider employing a mixed methods study design, incorporating cross-sectional analytical methods such as surveys. This approach can be complemented by the exploration of the participants' experiences and knowledge, providing a more comprehensive understanding. Furthermore, ensuring the inclusion of large and representative samples is crucial for robust findings. Researchers might also opt for more advanced study designs, such as longitudinal or qualitative approaches, to delve deeper into the subject matter. Additionally, to capture a broader and more diverse perspective within Saudi Arabia, conducting multicenter studies across various regions and populations would contribute to a more comprehensive and nuanced understanding of the topic.

## Conclusions

This study provides valuable insights into the awareness, knowledge, and acceptance of the herpes zoster vaccine among Saudi Arabian citizens in Al-Ahsa. The findings indicate a relatively high level of awareness and knowledge about the vaccine, as well as a favorable attitude toward its effectiveness and availability. However, the vaccination rate remains low. The study highlights the need for targeted education and awareness campaigns, especially among healthcare providers, to address misconceptions and improve vaccine uptake. Moreover, efforts should focus on promoting vaccination among the recommended target populations, including individuals above 50 years and immunocompromised patients. Addressing barriers to vaccination, such as the perception of being in good health, is crucial for increasing vaccine coverage.
